# H and HL synergistically regulate jasmonate-triggered trichome formation in tomato

**DOI:** 10.1093/hr/uhab080

**Published:** 2022-01-20

**Authors:** Bing Hua, Jiang Chang, Xiaoqian Han, Zhijing Xu, Shourong Hu, Shuang Li, Renyin Wang, Liling Yang, Meina Yang, Shasha Wu, Jingyuan Shen, Xiaomin Yu, Shuang Wu

**Affiliations:** College of Horticulture, FAFU-UCR Joint Center for Horticultural Biology and Metabolomics, Fujian Agriculture and Forestry University, Fuzhou 350002, China; College of Horticulture and Plant Protection, Yangzhou University, Yangzhou 225009, China; College of Horticulture, FAFU-UCR Joint Center for Horticultural Biology and Metabolomics, Fujian Agriculture and Forestry University, Fuzhou 350002, China; College of Horticulture, FAFU-UCR Joint Center for Horticultural Biology and Metabolomics, Fujian Agriculture and Forestry University, Fuzhou 350002, China; College of Horticulture, FAFU-UCR Joint Center for Horticultural Biology and Metabolomics, Fujian Agriculture and Forestry University, Fuzhou 350002, China; College of Horticulture, FAFU-UCR Joint Center for Horticultural Biology and Metabolomics, Fujian Agriculture and Forestry University, Fuzhou 350002, China; College of Horticulture, FAFU-UCR Joint Center for Horticultural Biology and Metabolomics, Fujian Agriculture and Forestry University, Fuzhou 350002, China; College of Horticulture, FAFU-UCR Joint Center for Horticultural Biology and Metabolomics, Fujian Agriculture and Forestry University, Fuzhou 350002, China; College of Horticulture, FAFU-UCR Joint Center for Horticultural Biology and Metabolomics, Fujian Agriculture and Forestry University, Fuzhou 350002, China; College of Horticulture, FAFU-UCR Joint Center for Horticultural Biology and Metabolomics, Fujian Agriculture and Forestry University, Fuzhou 350002, China; College of Horticulture, FAFU-UCR Joint Center for Horticultural Biology and Metabolomics, Fujian Agriculture and Forestry University, Fuzhou 350002, China; College of Horticulture, FAFU-UCR Joint Center for Horticultural Biology and Metabolomics, Fujian Agriculture and Forestry University, Fuzhou 350002, China; College of Horticulture, FAFU-UCR Joint Center for Horticultural Biology and Metabolomics, Fujian Agriculture and Forestry University, Fuzhou 350002, China; College of Horticulture, FAFU-UCR Joint Center for Horticultural Biology and Metabolomics, Fujian Agriculture and Forestry University, Fuzhou 350002, China

## Abstract

The development of trichomes, which protect plants against herbivores, is affected by various stresses. In tomato, previous studies showed that stress-triggered jasmonic acid (JA) signaling influences trichome formation, but the underlying mechanism is not fully resolved. Here, we found that two C2H2 zinc finger proteins synergistically regulate JA-induced trichome formation in tomato. The naturally occurring mutations in the *H* gene and its close homolog *H-like* in a spontaneous mutant, LA3172, cause severely affected trichome development. Compared with the respective single mutant, the *h*/*hl* double mutant displayed more severe trichome defects in all tissues. Despite their partially redundant function, the *H* and *HL* genes regulate trichome formation in a spatially distinct manner, with *HL* more involved in hypocotyls and leaves while *H* is more involved in stems and sepals. Furthermore, the activity of H/HL is essential for JA-triggered trichome formation. The JA signaling inhibitor SlJAZ2 represses the activity of H and HL via physical interaction, resulting in the activation of THM1, a negative regulator of trichome formation. Our results provide novel insight into the mechanism of trichome formation in response to stress-induced JA signaling in tomato.

## Introduction

Trichomes are formed in all terrestrial plants, serving as a protective layer against various stresses [1–3]. In tomato, there are seven types of trichomes, of which types II, III, and V are non-glandular trichomes, while all the rest are glandular trichomes [4]. These trichomes exhibit distinct morphology: types I and IV have unicellular glands, and VI and VII have multicellular glands; types I and II (here called long trichomes) have multicellular bases and usually are 2–3 mm long with 6–10 stalk cells; types IV–V contain unicellular bases and the stalks are ~0.1–0.2 mm long with two to four stalk cells [4–7]. The diverse forms of trichomes not only form physical barriers but also produce chemicals, including acyl sugars and terpenoids, to enhance insect resistance in tomato [1, 2, 6, 8].

Jasmonic acid (JA), a plant stress hormone, mediates the resistance of plants against mechanical stress, pest attack, and pathogenic infection [9–17]. The biosynthesis of JA and the expression of JA signaling genes such as *MYC1* and *MYC2* are activated upon pest attack [16, 18–20]. However, JA-Ile signaling can also be hijacked by pathogenic bacteria via released effectors to antagonize salicylic acid-mediated plant immunity [21–25]. In addition, JA plays important roles in a variety of developmental processes, including flowering, root hair development, and trichome formation [26–28]. Previous studies showed that JA mediated herbivore-resistance traits in tomato by promoting trichome formation [21, 29–32]. A group of inhibitory proteins called JASMONATEZIM DOMAIN (JAZ) and the general co-repressor TOPLESS physically interact to form the repression complex of JA signaling [33, 34]. In the presence of JA, JAZ proteins undergo ubiquitin-mediated degradation in an SCF^COI1^-dependent manner [11, 26, 35–38]. JAZ repressors physically bind to a range of downstream transcription factors [14, 17, 39–41]. In addition, the associated TOPLESS (TPL) proteins can epigenetically repress transcription factors via recruiting histone-modifying enzymes and other chromatin-remodeling factors [42, 43]. In lateral root formation, for instance, TPL represses Auxin response factor 7 (ARF7) activity via interaction with indole 3-acetic acid 14 (IAA14) [44]. TPL also functions in JA signaling by forming a repressive complex with JAZ to block the activity of MYC2 [43, 45]. TOPLESS-RELATED 2 (TPR2) was shown to suppress jasmonate-responsive anthocyanin accumulation by repressing the WD-repeat/bHLH/MYB complex [46]. Similarly, TPL was also reported to repress the activity of MYB75 and anthocyanin accumulation via interaction with HOMEOBOX ARABIDOPSIS THALIANA 1 (HAT1) [47]. When plants are challenged by stresses, large amounts of active JA-Ile are produced, which promotes the formation of JA-Ile-dependent co-receptors, including CORONATINE-INSENSITIVE1 (COI1, an F-box subunit), Skp1, Cullin, and Rbx1 proteins [11, 17, 35–37, 48–51]. This SCF ^COI1^ ubiquitin ligase leads to the degradation of JAZ repressors via 26S proteasomes, thereby releasing the transcriptional activity of JAZ-bound transcription factors [11, 17, 35–37, 51, 52]. So far, a wide range of transcription factors has been found to be inhibited by JAZ proteins, which includes MYB (MYB21, MYB24, GhMYB25-like, and OsMYB30), bHLH (MYC2, MYC3, MYC4, and SlMYC1), HD-ZIP IV (GhHOX1, AnHD1, AnHD8, Woolly, and SlHD8), and DELLA [14, 17, 33, 39–41, 48–50, 53–61]. During trichome formation in *Arabidopsis*, JAZ proteins interact with the WD-repeat/bHLH/MYB complex, the core regulatory module of trichome initiation [62]. In cotton, GhJAZ2 negatively affects fiber initiation by binding to the GhMYB25-like transcription factor [57]. In tomato, the loss of function of COI1, an F-box protein that is required for JA signaling, jeopardizes the formation of type VI trichomes [9]. In addition, SlJAZ2 can suppress Woolly, a key transcription factor regulating trichome formation in tomato [60, 63, 64]. Although evidence supporting the involvement of JA signaling in trichome formation has been accumulating, several downstream elements are not identified and the underlying mechanism is still not fully resolved.

Previous studies have shown that a large number of transcription factors, many of them belonging to the C2H2 zinc finger protein (ZFP) family, are involved in trichome formation in multiple species [65, 66]. In *Arabidopsis*, GLABROUS INFLORESCENCE STEMS (GIS), GIS2, ZINC FINGER PROTEIN5 (ZFP5), and ZINC FINGER PROTEIN8 (ZFP8) regulate trichome formation through GL1, an MYB-like protein [67–70]. In cucumber, a C2H2 zinc finger protein called Tu regulates spine (a special type of trichome) formation by promoting cytokinin biosynthesis [71]. Hair, a C2H2 zinc finger protein, regulates the formation of multicellular trichomes in tomato [72]. However, the relationship between C2H2–ZFP-mediated regulation and JA signaling is still unclear.

In this study, we cloned a homolog of *H* (*Hair*), named *Hair-like* (*HL*), that positively regulates the formation of long trichomes in tomato. Loss of function of both H and HL resulted in the absence of long trichomes in all tissues. We further found H/HL function is essential for JA-induced trichome formation, in which H/HL activity is usually repressed by JAZ2. In the presence of JA, high H/HL activity represses the expression of *THM1*, an R2R3 MYB transcription factor that negatively regulates trichome formation. Therefore, our results uncovered a new mechanism mediated by H and HL in JA-induced trichome formation in tomato.

## Results

### JA-induced trichome formation is undermined in LA3172

**Figure 1:**
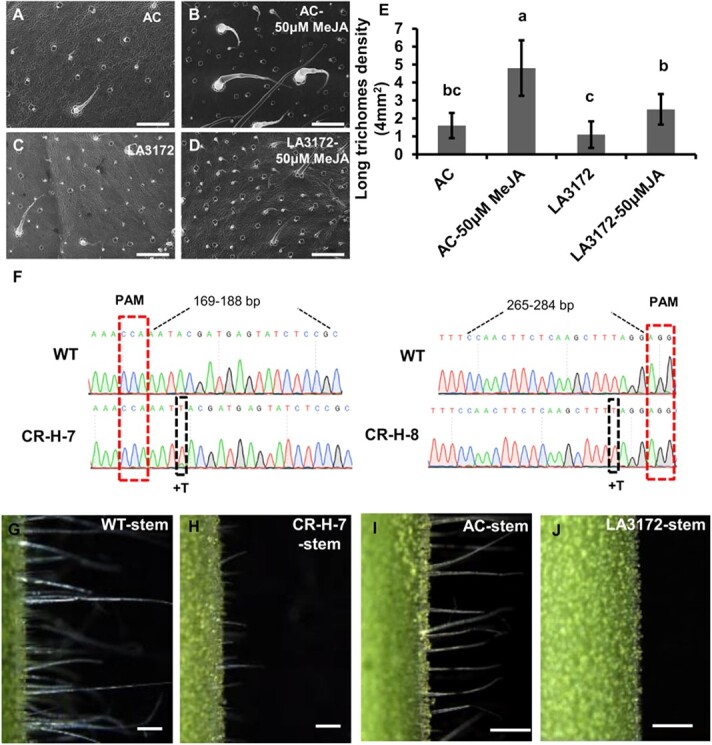
H is required for JA-induced trichome development. **a**–**d** Trichome phenotype on leaves of AC (**a**, **b**) and LA3172 (**c**, **d**) by SEM. Bars: 500 μm. **a**, **c** control (CK); **b**, **d** MeJA treatment. **e** Quantitative analysis of long trichome density on leaves of AC and LA3172 with or without 50 μM MeJA treatment. Error bars represent the standard deviation. Different letters denote significant differences (*P* < .05) from Fisher’s LSD (least significant difference) test after ANOVA. **f** Sequence analysis of the *H*^CR^ line generated by CRISPR/Cas9. A single nucleotide T is inserted in the CR-H-7 and CR-H-8 lines. The red dashed box shows the protospacer adjacent motif (PAM); the black dotted box shows the mutant nucleotide; the black dashed lines show the target site. **g**–**j** Trichome phenotype on stems of WT (**g**), *h* mutant (**h**), AC (**i**), and LA3172 (**j**). Bar (**g**–**j**) = 1 mm.

A previous study showed that the mutation of H in LA3172 [a spontaneous mutant derived from ‘Ailsa Craig’(AC)] resulted in fewer trichomes [72]. As JA promotes trichome formation in tomato, we tested whether H is involved in JA-mediated trichome formation. We treated LA3172 and AC [wild type (WT) of LA3172] with 50 μM methyl jasmonate (MeJA) once a week and examined the density of long trichomes (types I + II) after 1 month. Consistent with previous studies, our scanning electron microscopy (SEM) observations clearly showed that the density of long trichomes in AC was notably increased with JA treatments (Fig. 1a, b, e) [9, 32]. However, in the LA3172 background, we observed markedly reduced induction of long trichomes after JA treatment (Fig. 1c, d, e). To verify the effectiveness of JA treatments, we quantified terpene content by gas chromatography–mass spectroscopy (GC–MS) in all JA-treated tomatoes. We detected significantly increased levels of terpenes, including four monoterpenes (α-pinene, 2-carene, α-phellandrene, and β-phellandrene) and two sesquiterpenes (β-caryophyllene and α-humulene), after 50 μM MeJA treatment (Supplementary Fig. 1). This result suggested that the MeJA treatment was effective, and the mutations in LA3172 can block JA-induced trichome formation.

As LA3172 is a naturally occurring mutant in which many spontaneous mutations could take place, we further verified the previous results by generating a single mutant of the *H* gene using CRISPR/Cas9. We chose sequences 169-188 and 265-284 bp downstream of the transcription initiation sequence of *H* as the target site, and obtained the CR-H-7 and CR-H-8 lines with a nucleotide (T) insertion that led to premature termination (Fig. 1f; Supplementary Fig. 2A-B). Unexpectedly, there were still many long trichomes on the stems of the *h* mutant, which questioned the involvement of the *H* gene in the phenotype of the LA3172 mutant (Fig. 1g–j; Supplementary Fig. 3). Hence, we speculated that other genes might be involved in conferring the trichome phenotype in LA3172.

### Defect in HL promoter is correlated with reduced *HL* expression in LA3172

Many trichome-regulating C2H2 ZFPs from *Arabidopsis* and tomato are clustered in one group [73]. There is another C2H2 zinc finger protein (Solyc10g078990) in the cluster of *H* in tomato [73]. Interestingly, the gene is very close to H on chromosome 10 and the interval between the two genes is only ~25 000 bp.

**Figure 2:**
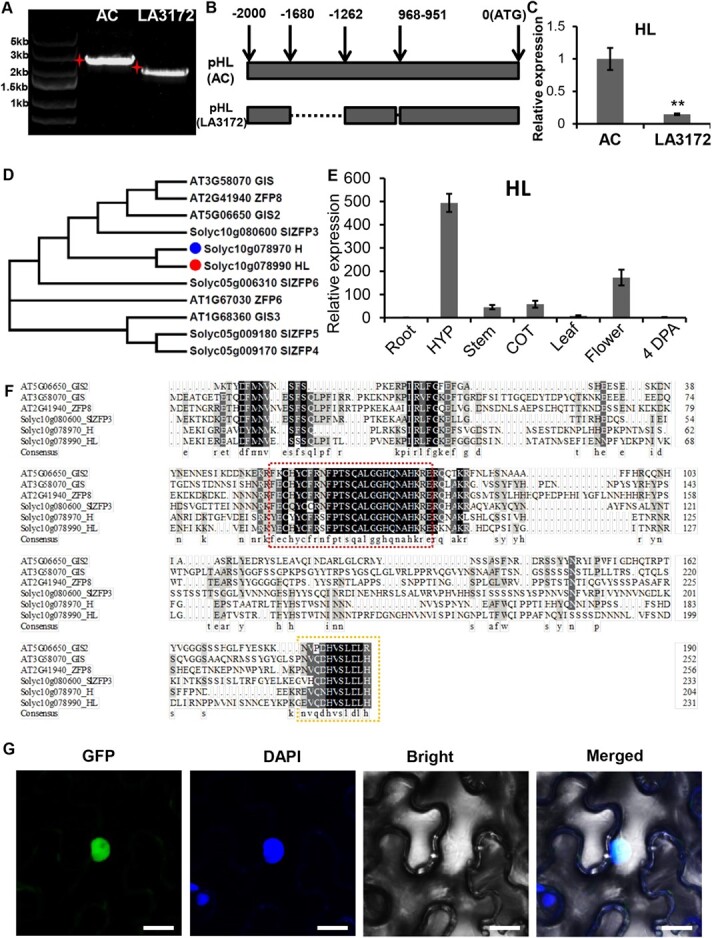
The HL promoter is defective and *HL* is down-regulated in LA3172. **a** The promoter of HL in LA3172 was shorter than that in AC shown by the agarose gel electrophoresis (AGE). The left lane shows the DNA marker; the middle lane shows the HL promoter of AC; the right lane shows the HL promoter of LA3172. **b** The genomic diagram shows the sequencing result of the HL promoter in AC and LA3172. Black arrows show the genomic location. The upper lane shows the *HL* promoter of AC; the lower lane shows the HL promoter of LA3172. The dotted line shows the deletion fraction of the HL promoter in LA3172. **c** The transcriptional level of HL analyzed in LA3172 and AC by qRT–PCR. The bars represent the standard deviation. Asterisks indicate significant difference according to Fisher’s LSD (least significant difference) test after ANOVA (^**^P < .01). **d** Phylogenetic analysis of H and its closely related proteins in tomato and *Arabidopsis thaliana*. The amino acid sequences of five genes from tomato and *A. thaliana* were used to construct the phylogenetic tree using MEGA6. The red dot indicates HL and the blue dot indicates H. **e** Tissue-specific expression of *HL* by qRT–PCR. Root, hypocotyl (HYP), stem, cotyledon (COT), leaf, flower, and 4 days post-anthesis fruit (4 DPA) were used for the expression analysis. **f** Protein sequence alignment of GIS, ZFP8, GIS2, SlZFP3, H, and HL by DNAMAN. The red frame shows the C2H2 domain and the yellow frame shows the conserved C-terminal domain of those proteins. **g** Subcellular localization of HL-GFP. The GFP signal overlaps with the DAPI signal. Bars: 25 μm.

We compared the coding sequence (CDS) of HL between LA3172 and AC. There were only a small number nonsense mutations in LA3172 (Supplementary Fig. 4A-B). We further amplified the sequence 2251 bp upstream from the transcription initiation sequence of HL by PCR. The results showed that the band of the HL promoter in LA3172 was smaller than that in AC, indicating a potentially shorter HL promoter in LA3172 (Fig. 2a). The sequencing showed that there were two fragment deletions and several SNPs in the HL promoter of LA3172 (Fig. 2b; Supplementary Fig. 5). To test the effect of these mutations, we examined the expression level of *HL* in both AC and LA3172 by quantitative real-time reverse transcription–PCR (qRT–PCR). Our result revealed that *HL* expression was dramatically down-regulated in LA3172 (Fig. 2c).

**Figure 3:**
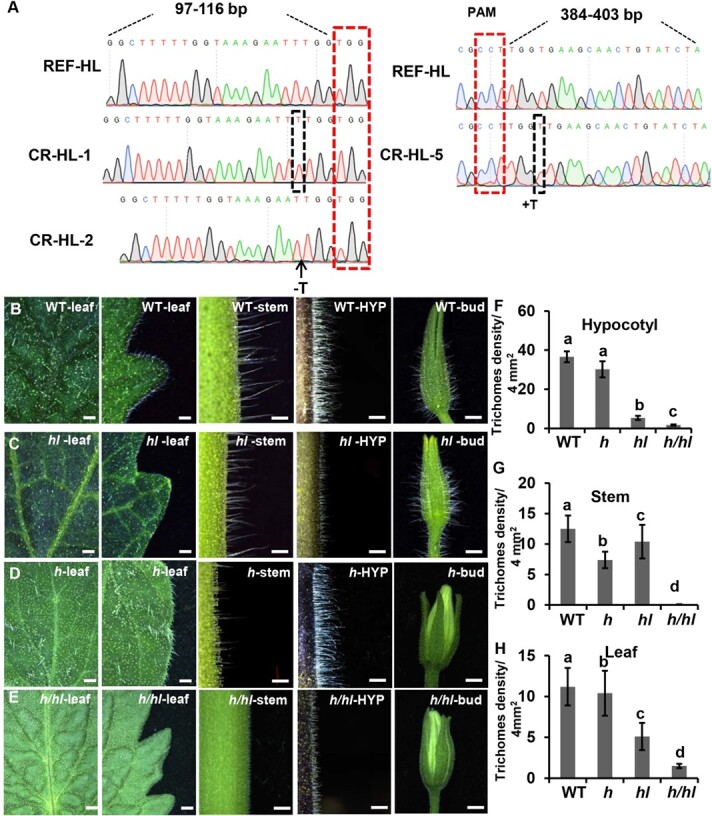
The trichome phenotypes of WT, *h* mutant, *hl* mutant, and *h*/*hl* double mutant. **a** Sequence analysis of *HL*^CR^ lines. REF represents the reference sequence of the target site. There is a T insertion in the CR-HL-1 and CR-HL-5 lines and a T deletion in the CR-HL-2 line. Red dotted boxes show the protospacer adjacent motif (PAM); black dotted boxes show the mutant nucleotide; the black dotted lines show the target site. **b**–**e** The trichome phenotype of WT (**b**), *hl* mutant (**c**), *h* mutant (**d**), and *h*/*hl* mutant (**e**) under a stereomicroscope. Bars (**b**–**e**): 1 mm. **f**–**h** Quantitative analysis of the long trichomes on the hypocotyl (**f**), stem (**g**), and leaf (**h**). The error bars represent the standard deviation. Different letters denote significant differences (*P* < .05) from Fisher’s LSD (least significant difference) test after ANOVA.

We then reconstructed the phylogenetic tree of all homologous genes of *H* in tomato and *Arabidopsis*. The result showed that Solyc10g078990 was indeed the closest homologous gene to *H* in tomato, so we named it *Hair-like* (*HL*) (Fig. 2d). In addition to the C2H2 domain, there are 11 amino acids in the C-terminal region that appeared to be conserved in these proteins (Fig. 2f). We further examined the tissue expression pattern of *HL* using qRT–PCR. As shown in Figure 2e, *HL* had the highest transcriptional level in hypocotyls (Fig. 2e). HL-GFP (green fluorescent protein) fusion protein showed nuclear localization in tobacco leaves (Fig. 2g), suggesting its potential role as a transcription factor.

### H and HL regulate trichome formation in a spatially distinct manner

To test HL’s function, we generated the *hl* mutant using CRISPR/Cas9. We selected two different target sites for CRISPR/Cas9, and obtained a number of *HL* mutants: the CR-HL-1 and CR-HL-5 lines had a nucleotide insertion (T) and the CR-HL-2 line had a nucleotide deletion (T), leading to a frame-shift mutation and premature termination, respectively (Fig. 3a; Supplementary Fig. 6A–B). We then observed the trichomes on hypocotyls, stems, leaves, and sepals in the WT and the *hl* mutant. We found that the long trichomes on the hypocotyls and leaves of the *hl* mutant were substantially less than those in WT (Fig. 3b, c). We further quantified the density of long trichomes by SEM (Supplementary Fig. 7A-B). The result showed that the density of long trichomes in the *hl* mutant was decreased by ~90 and 60% on hypocotyls and leaves compared with WT (Fig. 3f, h). However, the long trichomes on the stems of the *hl* mutant exhibited no significant difference from those in WT (Fig. 3g; Supplementary Fig. 7A-B).

Different from the *hl* mutant, the density of long trichomes on stems and sepals was significantly reduced in the *h* mutant (Fig. 3b, d; Supplementary Fig. 7A, C). The quantitative analysis showed that the density of long trichomes in the *h* mutant was decreased slightly on HYP and leaves, and significantly decreased on stems and sepals (Fig. 3f–h). Therefore, the H and HL transcription factors seemed to function in a spatially distinct manner to regulate trichomes.

To characterize the genetic relationship between H and HL, we generated the *h*/*hl* double mutant (Fig. 3e; Supplementary Fig. 7D). Interestingly, the double mutant exhibited more severe defects than either the *h* or *hl* single mutant. The long trichomes on hypocotyls, stems, leaves, and sepals nearly disappeared in the *h*/*hl* double mutant (Fig. 3c–e; Supplementary Fig. 7B-D). This observation was verified by the quantitative analysis (Fig. 3b, d, f–h). Therefore, H and HL also seemed to function synergistically in regulating tomato trichomes.

### JA-induced trichome formation relies on H/HL activity

**Figure 4:**
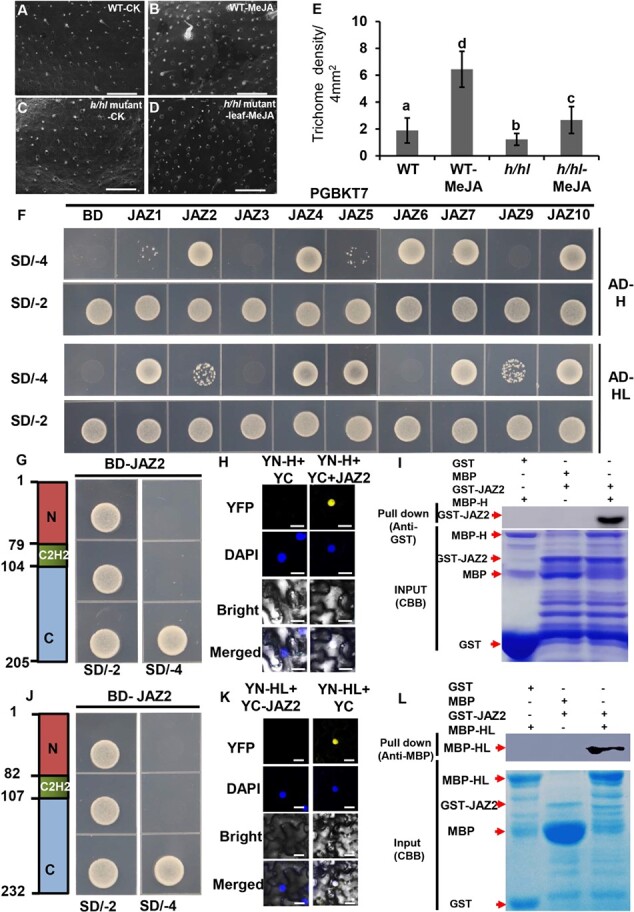
JAZ2 physically interacts with H and HL. **a**–**d** Trichome phenotype on the leaves of WT (**a**, **b**) and *h*/*hl* mutant (**c**, **d**) with or without 1 mM MeJA treatment under SEM. Bars: 500 μm. **a**, **c** Cytokinin treatment; **b**, **d** MeJA treatment. **e** Quantitative analysis of long trichome density on the leaves of WT and *h*/*hl* mutant with or without MeJA treatment. The error bars represent the standard deviation. Different letters denote significant differences (*P* < .05) from Fisher’s LSD (least significant difference) test after ANOVA. **f** Interactions between H/HL and JAZs are detected by Y2H assays. JAZs are fused with the GAL4 DNA binding domain in pGBKT7 vector (BD-JAZs); H or HL is fused with the GAL4 activation domain in pGADT7 vector (AD-H; AD-HL). The first and third rows show transformed yeast cells cultured on SD/−T-L-H-A (SD/−4) medium and the second and fourth rows show transformed yeast cells cultured on SD/−T-L (SD/−2) medium. **g**, **j** Y2H assays examining interactions between C-terminal of H (**g**)/HL (**j**), and JAZ2. The left diagrams show the three domains of H and HL. Numbers show the amino acid positions of the three domains. The red boxes show the N-terminal domain (marked N); the green boxes show the C2H2 domain; the gray boxes show the C-terminal domain (marked C) of H (**g**, **j**). All transformants were incubated on SD/−T-L (SD/−2) and SD/−T-L-H-A (SD/−4) medium. **h**, **k** BiFC analysis shows that H (**h**) and HL (**k**) physically interact with JAZ2. H and HL are fused into pUC-SPYNE vector (YN-H; YN-HL); JAZ2 is fused into pUC-SPYCE vector (YC-JAZ2). These vectors are transformed into *A. tumefaciens* GV3101. Tobacco injection was conducted in the following combinations: YN-H/YN-HL with YC-JAZ2, and YN-H/YN-HL with YC. YN-H/YN-HL and YC was used as the negative control. Bar: 50 μm. The YFP signal is found in the combination of YN-H with YC-JAZ2 (**h**) and YN-HL with YC-JAZ2 overlapped with the DAPI signal (**k**), but not in the combination of YN-H with YC. **i**, **l***In vitro* pull-down assays testing for the interaction between MBP-H/MBP-HL and GST-JAZ2. The upper panels show the result of pull-down and the panels below show the result of input by Coomassie brilliant blue staining. Protein samples were immunoprecipitated with anti-GST antibody (**i**) or anti-MBP (**l**). MBP protein and GST protein were used as negative controls (left and middle lanes) (**i**, **l**). GST-JAZ2 protein was not pulled down by MBP (left lane); GST was not pulled down by MBP-H (middle lane); GST-JAZ2 protein could be pulled down by MBP-H (right lane) (**i**). MBP protein was not pulled down by GST-JAZ2 (left lane); MBP-HL was not pulled down by GST (middle lane); MBP-HL protein could be pulled down by GST-JAZ2 (right lane) (**l**). The red arrows indicate the specific bands of different purified proteins.

As JA-induced trichome formation was undermined in LA3172, it is likely that both H and HL participate in JA signaling. To test this hypothesis, we treated the *h*/*hl* mutant with 1 mM MeJA once a week and examined the density of trichomes after 1 month. Under SEM, we visualized a clear increase in the density of long trichomes in WT with MeJA treatment (Fig. 4a, b, e), while the effect of MeJA treatment on trichome density was markedly lower in the *h*/*hl* double mutant (Fig. 4c, d, e).

We then examined whether H and HL are repressed by JAZs of tomato. First, we tested the protein–protein interaction between H/HL and JAZs using yeast two-hybrid (Y2H) assays. We fused the CDS of JAZs with GAL4 DBD (the DNA-binding domain) in pGBKT7 (BD) and the CDS of H or HL with GAL4 AD (the activation domain) in pGADT7 (AD). The combination of H/HL-AD and BD empty vectors was used as a negative control. After incubation on SD/−4 medium for 4 days, both H and HL showed a positive interaction with multiple JAZ proteins (Fig. 4f). Among these JAZs, JAZ2 was previously reported to inhibit trichome formation when overexpressed in tomato [64]. We thus focused on the interaction between H/HL and JAZ2. To screen the exact interacting domain of H/HL, we cloned the separate domains, including the N- terminus, C2H2 domain, and C-terminus. We found that interaction occurred between the domain of the C-terminus of H/HL and JAZ2 (Fig. 4g, j). To verify the interaction, we conducted bimolecular fluorescence complementation (BiFC) and *in vitro* pull-down assays. We cloned the CDS of H or HL into pUC-SPYNE vector (named YN-H or YN-HL) and the CDS of JAZ2 into pUC-SPYCE vector (YC-SlJAZ2). The combinations of empty pUC-SPYCE vector with YN-H or YN-HL were used as a negative control. A yellow fluorescent protein (YFP) fluorescence signal was clearly visible in tobacco leaves co-infiltrated with YN-H or YN-HL combined with YC-SlJAZ2, but not in the negative control (Fig. 4h, k). We further used purified maltose binding protein (MBP)-tagged H (MBP-H) and glutathione *S*-transferase (GST)-tagged JAZ2 (GST-JAZ2) to perform pull-down assays. The combination of MBP-H with GST or MBP with GST-JAZ2 was used as a negative control. MBP-H, but not MBP, could pull down GST-JAZ2 with high efficiency (Fig. 4i). Using the same strategy, we detected the efficient pull-down of MBP-HL by GST-JAZ2 (Fig. 4l).

### H and HL directly repress *THM1* expression

**Figure 5:**
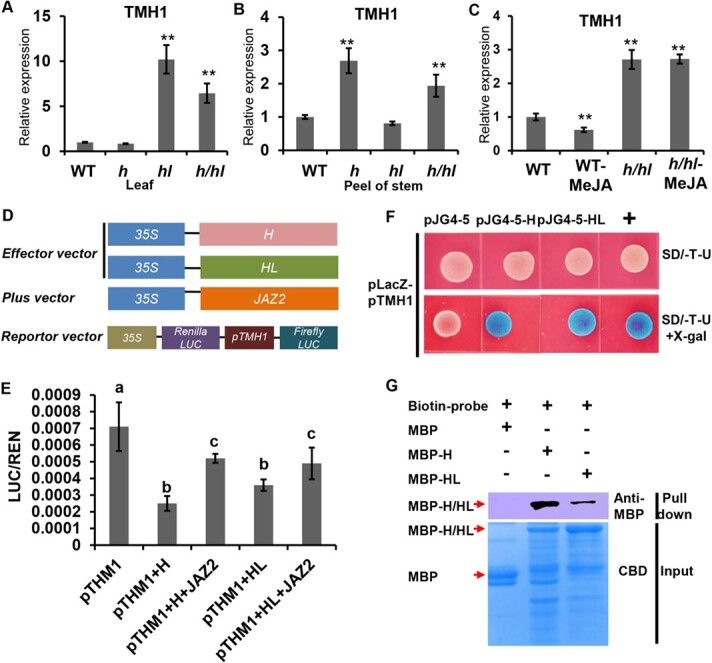
THM1 is the direct target of H and HL. **a** qRT–PCR assays show that *THM1* expression level is increased in leaves of the *hl* mutant and *h*/*hl* double mutant, but not in the leaves of the *h* mutant. **b** qRT–PCR assays show that *THM1* expression level is increased in the stems of the *h* mutant and *h*/*hl* double mutant, but not in the *hl* mutant. **c** Expression level of *THM1* is decreased in WT but not in the *h*/*hl* double mutant after MeJA treatment for 12 hours. Asterisks mean a significant difference between the mutant and WT according to Fisher’s least significant difference (LSD) test after ANOVA (^**^P < .01). **d** Diagrams of the effector, plus vector and reporter vector used in dual-LUC assay. The CDSs of *H* and *HL* were fused into pXSN-Flag as effector vector. The CDS of JAZ2 was fused into pXSN-HA as plus vector. The promoter of THM1 (pTHM1) was fused with Firefly LUC reporter into pGREEN-0800 as effector vector. **e** Dual-LUC assay shows activation of the THM1 promoter by H and HL, which is repressed by JAZ2. Different letters denote significant differences (*P* < .05) from Fisher’s LSD (least significant difference) test after ANOVA. **f** Y1H assay showing the interaction between H/HL and THM1 promoter. The CDS of *H* or *HL* was inserted into the pJG4-5 vector (pJG4-5-H, pJG4-5-HL). *THM1* promoter was fused with the LacZ reporter gene in the pLacZ vector (pLacZ-pTHM1). The combination containing pJG4-5 and pLacZ-pTHM1 was used as the negative control. The combination of pJG4-5-H + pLacZ-pTHM1 and pJG4-5-HL + pLacZ-pTHM1 turns blue on SD/−Trp-Ura medium with X-gal. **g** Biotin-labeled DNA pull-down assay showing the interaction between MBP-H/MBP-HL and biotin-labeled THM1 promoter (biotin-pTHM1). The left lane shows biotin-pTHM1 does not pull down MBP; the middle lane shows that biotin-pTHM1 pulls down MBP-H; the right lane shows that biotin-pTHM1 pulls down MBP-HL.

In tomato, THM1, a R2R3 MYB transcription factor, acts as a negative regulator of long trichomes [74]. To explore the relationship between THM1 and H/HL, we examined the transcriptional level of *THM1* in mutants of *h*, *hl*, and *h*/*hl h*. The qRT–PCR detected the up-regulation of *THM1* in the leaves of the *hl* mutant and *h*/*hl* double mutant, but not in the *h* mutant (Fig. 5a). By contrast, the expression of *THM1* on stems increased in the *h* mutant and *h*/*hl* double mutant, but showed no significant difference in the *hl* mutant (Fig. 5b). These results suggested that the repression of THM1 by H and HL most likely occurred in a tissue-dependent manner. In addition, *THM1* expression was suppressed by MeJA treatment in WT, but not in the *h*/*hl* double mutant (Fig. 5c). To verify whether THM1 is the direct target of H and HL, we first conducted a dual-luciferase (LUC) reporter assay. We fused the ~2000 bp promoter of THM1 into the LUC reporter and expressed the CDS of H, HL, and JAZ2 in the pXSN-Flag vector driven by 35S as the effector (Fig. 5d). In the protoplast of *Nicotiana benthamiana*, the dual-LUC assay showed that the H and HL effectors successfully repressed the luminescence intensity, and the addition of JAZ2 weakened this repression by H and HL (Fig. 5e). To confirm the direct interaction, we performed the yeast-one hybrid (Y1H) assay via fusing the ~2000 bp promoter of *THM1* with LacZ reporter in the pLacZ (pTHM1-LacZ) vector, and tagged H or HL with B42 transcription activation domain-HA1 in the pJG4-5 vector (marked as pJG4-5-H or pJG4-5-HL in Fig. 5f). After being cultured on SD/−T-U medium with X-gal for 3 days, transformed yeast cells with pJG4-5-H or pJG4-5-HL became blue while cells with pJG4–5 remained white (Fig. 5f). To further verify the interaction, we conducted biotin pull down assays. We generated the biotin-tagged THM1 promoter (biotin-pTHM1) by PCR and incubated the MBP, MBP-H, or MBP-HL proteins with resin-bound biotin-pTHM1. The pull-down assay showed that MBP-H and MBP-HL proteins, but not MBP, could be detected (Fig. 5g). Based on these results, we conclude that H and HL directly regulate the expression of *THM1*.

## Discussion

**Figure 6:**
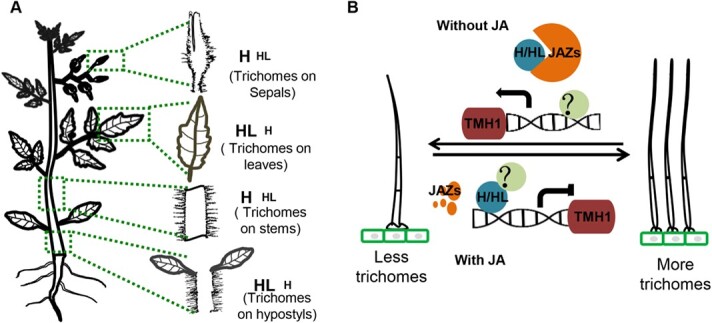
A proposed model of long trichome formation regulated by H/HL. **a** H/HL regulates long trichome formation on different tissues. The left diagrams show the hypocotyls, stems, leaves, and buds. The right diagrams show the different roles of H/HL in trichomes from different tissues: HL is mainly responsible for trichome formation on hypocotyls and leaves, while H is mainly responsible for trichome formation on stems and sepals. Large letters indicate the major effect and small letters indicate the minor effect. **b** Diagram showing H/HL directly represses *THM1* expression during JA-triggered trichome formation. When the JA level is high, the JAZ proteins are degraded and H/HL inhibits the transcription of *THM1*. H and HL directly bind to the *THM1* promoter, either blocking the potential uncharacterized transcriptional activators (marked ?) or inhibiting their ability for transcriptional activation. In the absence of JA, JAZ proteins bind to H/HL, releasing the repression of *THM1* to reduce long trichome formation.

Although H and HL transcription factors have a partial functional redundancy, they seem to act in a spatially distinct manner during trichome development. Apparently, the H transcription factor functions more in hypocotyls and leaves, while HL contributes more in stems and sepals (Fig. 6a). In addition, the function of H and HL seems to be indispensable for JA-triggered trichome formation.

### Trichome regulation is spatially distinct

Among the different tissues of plants, trichomes often display distinct morphology and distribution patterns. In *Arabidopsis*, trichomes are densely formed on the tissues, including leaves, stems, and sepals, but rarely on hypocotyls and cotyledons [75]. On leaves, trichomes usually have three or four branches, while the trichomes on stems and sepals have fewer or no branches [75]. In cottons, there are three types of trichomes on leaves and stems (multicellular capitate hairs, stellate hairs, and simple hairs), while the trichomes in seeds form single-cellular and unbranched fibers [76]. In tomatoes, type IV trichomes form mainly on cotyledons and juvenile leaves [77]. In this study, we demonstrated that more long trichomes are formed on hypocotyls and stems than on leaves (Fig. 3f–h). All these results support the idea that the regulation of trichome formation is presumably tissue-specific.

In line with this, several identified trichome regulators also function distinctly in different tissues. Knock-out of *Slwo* in tomato resulted in the reduction of type VII trichomes on leaves, but increased type VII trichomes on stems [60]. In this study, we found that the loss of function of H or HL caused a reduction of long trichomes on different tissues (Fig. 6a). This suggests that H and HL have a similar function, but the different spatial expression patterns presumably generate the tissue-specific activity of H and HL. In support of this, *HL* is highly expressed in hypocotyls, which probably accounts for the high density of long trichomes on hypocotyls (Fig. 2e). The lower expression level of *H* and *HL* on leaves could lead to the lesser formation of long trichomes on leaves (Fig. 2e; Supplementary Fig. 8 A–B) [72].

### The role of H and its homologs in trichome formation

Many members of the C2H2 zinc finger transcription factor family have been identified as regulators of trichomes in multiple species, including *Arabidopsis*, cucumber, and tomato. In this study, we identified a new C2H2 zinc finger transcription factor, named Hair-like (HL), that functions redundantly with H to regulate long trichome development. This is consistent with a recently published finding that HL promotes the formation and elongation of trichomes [78].

In *Arabidopsis*, GIS, GIS2, and ZFP8 are functionally equivalent proteins, which are activated by ZFP5 [70]. Trichome density shows no significant difference between *zfp5*/*zfp6* double mutants and *zpf5* (or *zfp6*) single mutants, indicating that ZFP5 and ZFP6 are largely redundant [79]. In tomato, however, the phenotype of trichomes in the *h*/*hl* double mutant was more severe than in *h* or *hl* single mutants, suggesting that the functions of H and HL are partially redundant. Besides, both qRT-PCR and the dual-LUC reporter assay showed that the expression of H was not significantly different in the hl mutant and the expression of HL was not significantly different in the h mutant (Supplementary Fig. 9A–F), suggesting that H and HL have no reciprocal regulation. These results indicate that the function of C2H2 ZFP proteins in trichome regulation is similar, but also distinct in many aspects. It is also puzzling that the trichome suppressors (H and HL) interact with the JA repressors (JAZ) to exert their functions. We speculate that there is another transcriptional activator that has not been identified yet, involved in the H/HL-mediated pathway. When a high level of JA removes JAZ proteins, it is possible that H/HL inhibits the transcription of *THM1* by competitively binding to the unknown transcriptional activator or by repressing its ability for transcriptional activation. When the JA level is low, JAZ proteins can compete with the unknown factor to bind to H/HL, releasing the repression of *THM1* expression (Fig. 6b). This mechanism involving competition for protein interaction by JAZs has already been reported by a number of studies [17, 60–80].

One concern regarding the generated CRISPR lines for *H* and *HL* genes is the potential restart of protein translation mediated by the downstream ATG of the target sites. We therefore analyzed the putative truncated proteins produced in these CRISPR lines. In all CRISPR lines with the *H* gene, we predicted four potential truncated proteins, among which was CR-H-7 with 1−57 aa left carries no functional domains (Supplementary Fig. 10A, C). Our quantification showed that CR-H-7 had the same phenotype as other CRISPR lines, implying that none of these CRISPR lines produce functional truncated proteins. However, in the case of HL CRISPR lines, we predicted six potential truncated proteins, and it seems that all truncated proteins cover the C2H2 domain (Supplementary Fig. 10B, D). Thus, it is possible that the putative truncated proteins in the *hl* mutant could still be functional.

### Zinc finger proteins mediate hormonal signaling in trichome formation

Many plant hormones, including gibberellins, cytokinins, JA, and salicylic acid, are involved in trichome formation [81–84]. In *Arabidopsis*, cytokinins and gibberellins were reported to activate the expression of *GIS*, *GIS2*, and *ZFP8* to regulate trichome initiation [67–69]. The expression of *ZFP6* is also induced by gibberellin and cytokinin treatments [79]. In cucumber, Tu was shown to promote cytokinin biosynthesis to regulate wart development [71]. In this study, we showed that JA promoted *HL* expression and H/HL was required for JA-induced trichome formation (Supplementary Fig. 8 C). In addition, JAZ2 can block the activity of H and HL in the repression of *THM1* expression. THM1 has been shown by previous studies to regulate auxin-induced trichome formation [74]. In the Y2H assay, H/HL could interact with multiple JAZ proteins (Fig. 4f), so JAZ2 may not be the only repressor of H/HL. Interestingly, the expression of *HL* seemed to be up-regulated by IAA, forming a feedback loop during trichome regulation [73]. In the *h*/*hl* mutant, the expression of *ARF3* and *ARF4* was decreased, but H/HL was not able to activate the expression of *ARF3* or *ARF4* promoter in the dual-LUC assay (Supplementary Fig. 11 A–D). Therefore, H and HL could be one of the intersections between auxin and JA signaling during the regulation of trichome formation.

## Materials and methods

### Plant materials and growth conditions

*Solanum lycopersicum* cv. ‘Alisa Craig’ (AC) and LA3172 were provided by the Tomato Genetics Resource Center (https://tgrc.ucdavis.edu). We obtained seeds of Micro-Tom from TOMATOMA (http://www.tomatoma.nbrp.jp/). Micro-Tom was used as WT. Tomato seeds were placed on moistened filter paper and germinated for 72 hours. Tomato plants were grown in a greenhouse under an 18/8 hours light/dark cycle. Tobacco (*N. benthamiana*) plants were grown in a greenhouse with controlled temperature and light (16 hours day/8 hours night; 22°C).

### Vector construction and plant transformation

For CRISPR-Cas9, we selected the target sites using the tool (http://skl.scau.edu.cn/targetdesign). Primers used for vector construction are listed in Supplementary Table 1. The target sites were cloned into the pTX vector using the Clone Express II One Step Cloning Kit (Vazyme Biotech C112-01/02). The vectors were transformed into *Agrobacterium tumefaciens* C58 competent cells. Plant transformation was conducted using the *A. tumefaciens*-mediated transformation method. We examined transgenic plants by sampling different parts of the plants, and then mixing them for PCR and sequencing. Homozygous lines were used for phenotypic and molecular characterization.

### Gene expression analysis

Samples used for gene expression were collected from 4-week-old plants. We peeled the exterior layers of stems or hypocotyls of tomatoes using tweezers, and collected them for RNA extraction using TransZol (TransGen Biotech ET101-01). A HiScript 1st Strand cDNA Synthesis Kit (Vazyme Biotech R111-01) was used for the synthesis of cDNA. AceQ qPCR SYBR Green Master Mix (Vazyme Biotech Q121-02) was used for qRT–PCR. The amplification was quantified using the CFX384 Real-Time System (Bio-Rad). For qRT–PCR, *SlACTIN2* (Solyc11g005330) was used as the internal control. Three biological replicates were performed for each experiment. Error bars in the figures represent the standard deviation of three biological replicates. Primers used for qRT–PCR are listed in Supplementary Table S1.

### Phylogenetic analysis

We selected the sequences of the six highest-scoring proteins of *Arabidopsis* using an H query in BLASTP. Similarly, the sequences of the six highest-scoring proteins of tomato were selected using the same BLASTP strategy on the Sol Genomics Network (https://solgenomics.net/). We then performed multiple sequence alignment with MEGA (version 6) and DNAMAN. We conducted the phylogenetic analysis using the neighbor-joining method.

### Microscopy observation

For trichome observation, we selected 4-week-old tomato seedlings for study. We performed quantitation on the second leaflet pair on the third leaf (the youngest leaf was counted as the first one and the oldest leaf as the last one). For the quantitation of trichomes on stems, the third internodes were selected for analysis (the internode next to the meristem was counted as the first one and the internode close to hypocotyls as the last one).

For light microscopy images, samples from 4-week-old plants were observed under a stereomicroscope (Leica DFC550). Samples from fresh plants were used for SEM (Environmental Scanning Electron Microscope TM3030 Plus, Hitachi, Japan) and the voltage was controlled at 15 kV. Six to 10 images were used for quantitative analysis.

### JA treatments

For qRT–PCR analysis, 4-week-old tomato plants were treated with 1 mM MeJA, and the samples were collected after 12 hours. To observe trichome density, we sprayed 1-week-old tomato plants with 50 μM MeJA once a week. Mature leaves were collected for trichome observation after 1 month.

### Analysis of volatile metabolites

The second leaflet pair of the third leaf (the youngest leaf was counted as the first one and the oldest leaf as the last one) was harvested from 5-week-old tomato plants (AC and LA3172), and then immersed in 1 ml of *n*-hexane containing 16 μg/ml tetradecane for 1 hour with gentle shaking at room temperature. The extract solution was analyzed with an Agilent 7890B gas chromatograph (Agilent Co., Santa Clara, CA, USA). A Pegasus HT time-of-flight mass spectrometer (LECO Co., Saint Joseph, MI, USA) (GC-TOF MS) was coupled with the gas chromatograph.

Compounds were separated on a capillary Restek Rxi^®^-5Sil MS capillary column (30 m × 0.25 mm × 0.25 μm film thickness) with a flow rate of 1 ml/minute. The injection volume was 1 μl and the injection temperature was 280°C. The compounds were isolated with splitless mode and the transfer line temperature was kept at 270°C. The column temperature was kept at 80°C for 2 minutes and then increased to 130°C at a rate of 10°C/minute for 2 minutes. Then, the column temperature was heated to 190°C at the rate of 5°C/minute and then heated to 250°C at the rate of 20°C/minute. The mass spectra ion source temperature was kept at 250°C. The electron energy was kept at 70 eV and the detector voltage was set to 1600 V. The scan range was 30–500 atomic mass units (AMU). The scan rate was 10 spectra/second acquisitions. The solvent delay time was set at 170 seconds. Compound contents were quantified using the internal standard. All compounds were determined by the comparison of retention times and were analyzed by mass spectra data of the National Institute of Standards and Technology and Wiley libraries (Agilent Technologies, Palo Alto, CA).

### Yeast-two hybrid assay

To construct the pGADT7-H vector, we amplified the CDS of H and fused it to GAL4 AD of the pGADT7 vector. The conserved domains of H were predicted by the Conserved Domain Search Service tool (https://www.ncbi.nlm.nih.gov/guide/domains-structures/). We amplified and fused three domains of H with GAL4 AD of the pGADT7 vector. We generated the pGADT7-HL using a similar strategy to that used for H. To generate pGBKT7-JAZs vectors, we amplified the CDSs of JAZs of tomato and fused them to the GAL4 DBD (DNA binding domain) of the pGBKT7 vector. We mixed the recombinant constructs and transformed them into yeast strain AH109. According to the manufacturer’s manual (Clontech), the Matchmaker GAL4 Two-Hybrid System was used for LiAc yeast transformation. The negative control was the combination of pGADT7 and pGBKT7-JAZs. SD-Leu-Trp (SD/−2) media were used for selecting the transformant. For scoring the interactions, transformants were diluted in H_2_O to 1 OD (OD600) and plated on SD/−Ade/-His/−Leu/−Trp (SD/−4) medium for 4 days.

### Bimolecular fluorescence complementation assay

To generate pUC-SPYCE-JAZ2 (CE-JAZ2) vector, we amplified the CDS of JAZ2 of tomato and cloned it to the C-terminal of YFP of the pUC-SPYCE vector. To generate pUC-SPYNE-H (NE-H) and pUC-SPYNE-HL (NE-HL), the full-length CDSs of H and HL were amplified and fused to the N-terminal of YFP in the pUC-SPYNE vector. *A. tumefaciens* GV3101 was used for the transformation of recombinant vectors. The combination of NE-H and pUC-SPYCE, pUC-SPYCE and NE-HL acted as the negative control. The *Agrobacterium* cells were mixed and subsequently co-transformed into leaves from 5-week-old *N. benthamiana*. After 48–72 hours of culture, we immersed the injected leaves in a DAPI (4′,6-diamidino-2-phenylindole) staining solution containing 1 μg/ml DAPI for 10 minutes, and washed them with water before observation under a confocal microscope. We observed the YFP and DAPI signals in tobacco leaves by confocal microscopy (LSM 880 Zeiss, Germany).

### Pull-down assay

To produce MBP-H and MBP-HL proteins, the full length CDSs of H and HL were amplified and fused to MBP in vector pMAL-c5X. To produce GST-JAZ2 protein, the full-length CDS of JAZ2 of tomato was amplified and fused to GST in vector pGEX-4 T-1. The recombinant vector was transformed into *Escherichia coli* strain Transetta (DE3). The expression of these proteins was induced by 0.125 mM isopropyl-β-d-thiogalactoside (IPTG). Amylose resin (NEB) was used for purification of MBP-H and MBP-HL proteins. GST Bind Resin (Novagen) was used for purification of GST-JAZ2 proteins. To detect H-JAZ2 interaction using *in vitro* pull-down assays, we incubated the GST-SlJAZ2 fusion protein with resin-bound MBP-H proteins for 2 hours with rotation at 4°C. For pull-down assays, MBP-HL proteins were incubated with GST Bind Resin-bound GST-SlJAZ2 fusion protein for 2 hours with rotation at 4°C. Purified GST and MBP proteins were used as the negative control. SDS–PAGE was used for protein separation and staining of polyacrylamide gels with Coomassie brilliant blue staining was used as a loading control. For immunoblot analysis, anti-GST antibody was used to detect the interaction with H-JAZ2, and anti-MBP antibody was used to detect the interaction with JAZ2-HL.

### Dual-luciferase activation assay

We conducted dual-LUC assays in N. *benthamiana* protoplasts as previously described [85, 86]. *N. benthamiana* plants were grown in an incubator kept under a light regime of 16 hours day/8 hours night at 22°C. The 2378-bp promoter region of the *THM1* gene was amplified and inserted into the vector pGREEN0800 (named pGREEN0800-pTHM1). We amplified the CDSs of H and HL and inserted them into the vector of pXSN-Flag (named Flag-H and Flag-HL). The full-length CDS of JAZ2 was amplified and inserted into the vector of pXSN-HA (named HA-JAZ2). The vectors of Flag-H and Flag-HL were used as effectors. HA-JAZ2 was used as the plus vector. pGREEN0800-pTHM1 was used as the reporter. Vector transformation of *N. benthamiana* protoplasts was performed as previously described [82]. LUC mix (Promega) was used to measure LUC activity with a luminometer (Cytation 5 Imaging Reader). We performed three biological replicates for each experiment. Error bars represent the SD of three biological replicates.

### Yeast one-hybrid assay

To generate the pLacZ-pTHM1 vectors, we amplified the 2378-bp promoter region of the *THM1* gene and fused it with the LacZ reporter gene in the pLacZ vector. To generate the pJG4-5-H and pJG4-5-HL vectors, we amplified the full-length CDSs of H and HL, and fused them with the B42 transcription activation domain-HA1 epitope in the pJG4-5 vector. The pJG4-5 and pLacZ-THM1 vectors acted as the negative control. To detect the interaction of H-pTHM1 and HL-pTHM1, we co-transformed the recombinant constructs into yeast strain EGY48. According to the manufacturer’s manual (Clontech), we performed yeast transformation using the Matchmaker GAL4 Two-Hybrid System. The co-transformed yeast cells were selected on SD/−Trp-Ura medium at 30°C for 3 days. To test for interaction, co-transformants were grown for 3 days on SD/−Trp-Ura + X-gal medium.

### Biotin pull-down assay

We performed the biotin pull-down experiment to verify the interaction of H or HL with the promoter of THM1. The biotin pull-down experiment was conducted as described previously [60, 87]. The forward and reverse primers used for the amplification of the THM1 promoter were labeled with biotin. The THM1 promoter was then amplified using PCR. The streptavidin–agarose bead (Streptavidin Agarose, Catalog Number SA100–04) was incubated with PCR products overnight at 4°C. MBP-H and MBP-HL fusion proteins were expressed and purified. We washed bead-bound DNA fragments with PBS buffer five times. After washing, bead-bound DNA fragments were incubated with the purified MBP-H or MBP-HL fusion proteins at 4°C. For immunoblotting, anti-MBP antibody was used for analysis of MBP-H or MBP-HL protein.

## Acknowledgements

This work was supported by the National Key Research and Development Program of China (2018YFD1000800), a grant from the National Natural Science Foundation of China (32000157), and the Natural Science Foundation of Fujian Province (2019J01379). We thank XiaXia Wang for assistance in the metabolomics analyses.

## Author contributions

B.H., J.C., and S.W. designed the experiments; B.H., J.C., X.H., Z.X., S.H., S.L., R.W., L.Y., J.S., and X.Y. performed most of the experiments and analyzed the data; M.Y. and S.W. performed tomato stable transformation; B.H., J.C. and S.W. wrote the article.

## Data availability

Sequence data from this article can be found in the Solgenomics databases (https://solgenomics.net/). Accession numbers: H, Solyc10g078970; HL, Solyc10g078990; JAZ1, Solyc07g042170; JAZ2, Solyc12g009220; JAZ3, Solyc03g122190; JAZ4, Solyc12g049400; JAZ5, Solyc03g118540; JAZ6, Solyc01g005440; JAZ7, Solyc11g011030; JAZ9, Solyc08g036640; JAZ10, Solyc08g036620; THM1, Solyc08g081500.

## Conflict of interest

The authors declare no competing interests.

## Supplementary data

Supplementary data is available at *Horticulture Research* online.

## Supplementary Material

Web_Material_uhab080
