# Supplementary material for: H and HL synergistically regulate jasmonate-triggered trichome formation in tomato
Source: Hortic Res. 2022 Jan 20;9:uhab080. doi: 10.1093/hr/uhab080 (PMC8973001; doi:10.1093/hr/uhab080)
Supplement: Web_Material_uhab080 [file web_material_uhab080.docx]

**
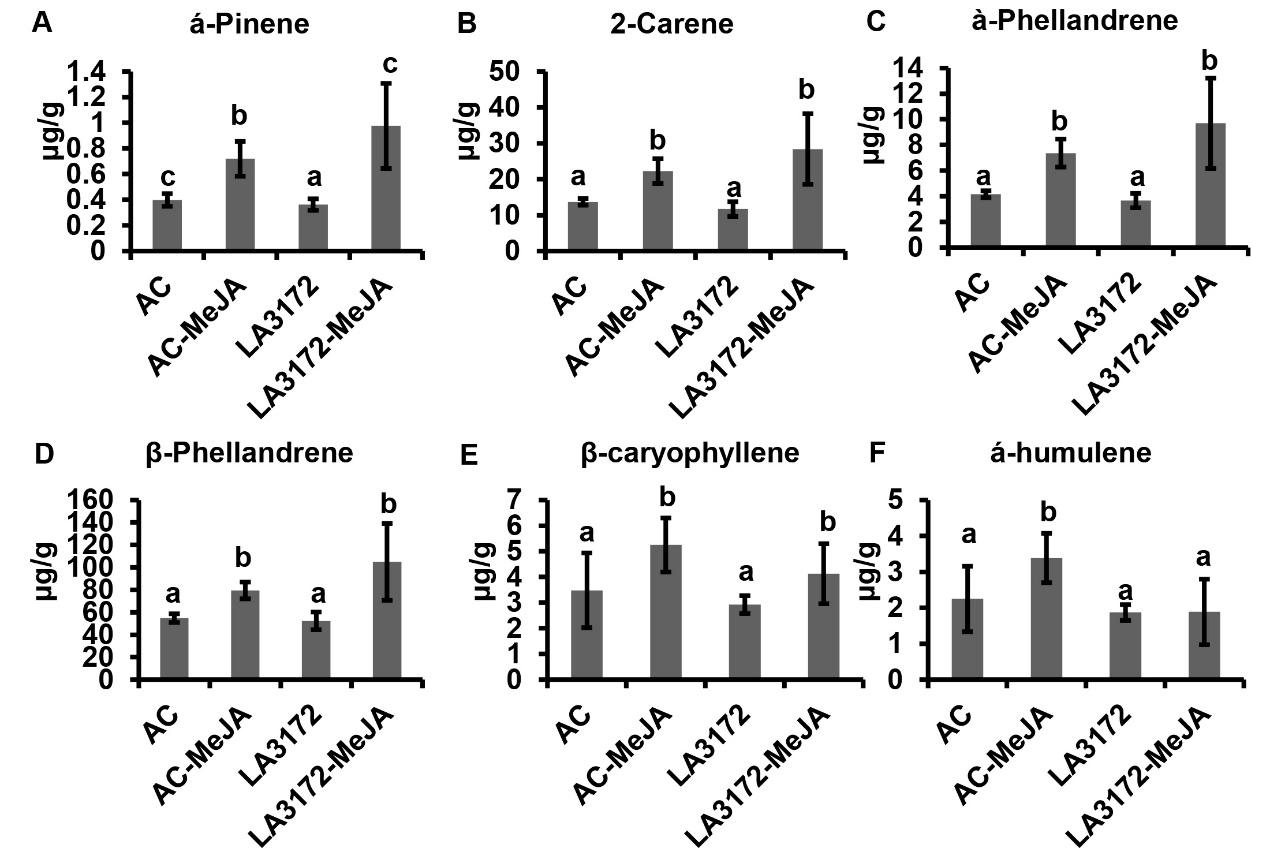
**

**Supplemental Fig. 1 MeJA treatment increases terpene synthesis.**

(A-F) The content of monoterpenes (à-Pinene, 2-Carene, α-Phellandrene, β-Phellandrene) and sesquiterpenes (β-Caryophyllene and α-humulene) in AC and LA3172 with or without 50 μM MeJA treatments. Different letters denote significant differences (P < 0.05) from Fisher’s LSD test after ANOVA.

**
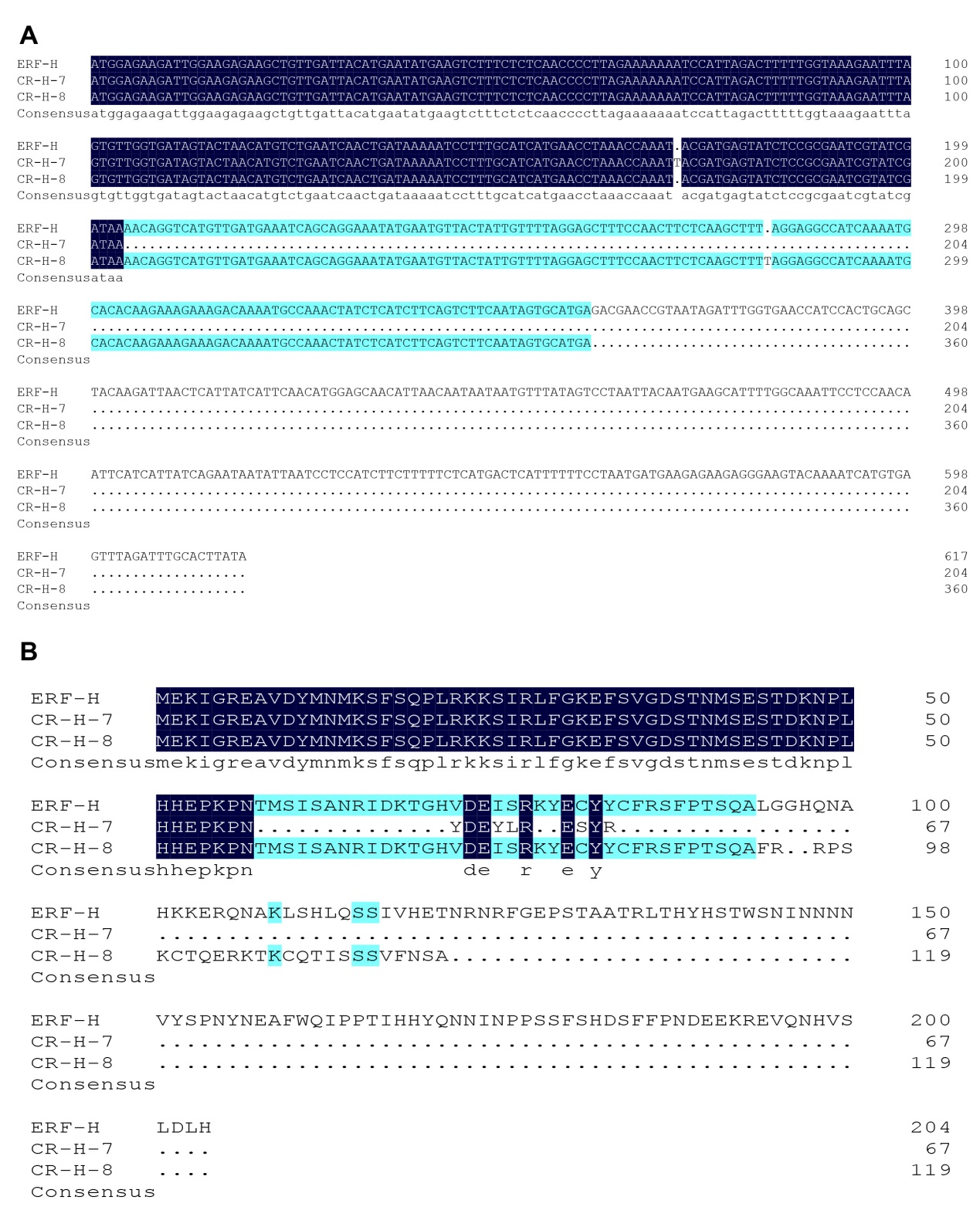
**

**Supplemental Fig. 2 The sequence analysis of H-CR lines generated by CRISPR/Cas9.**

(A) CDS alignment of H in CR-H plants. (B) Protein sequences alignment of H in CR-H plants.

**
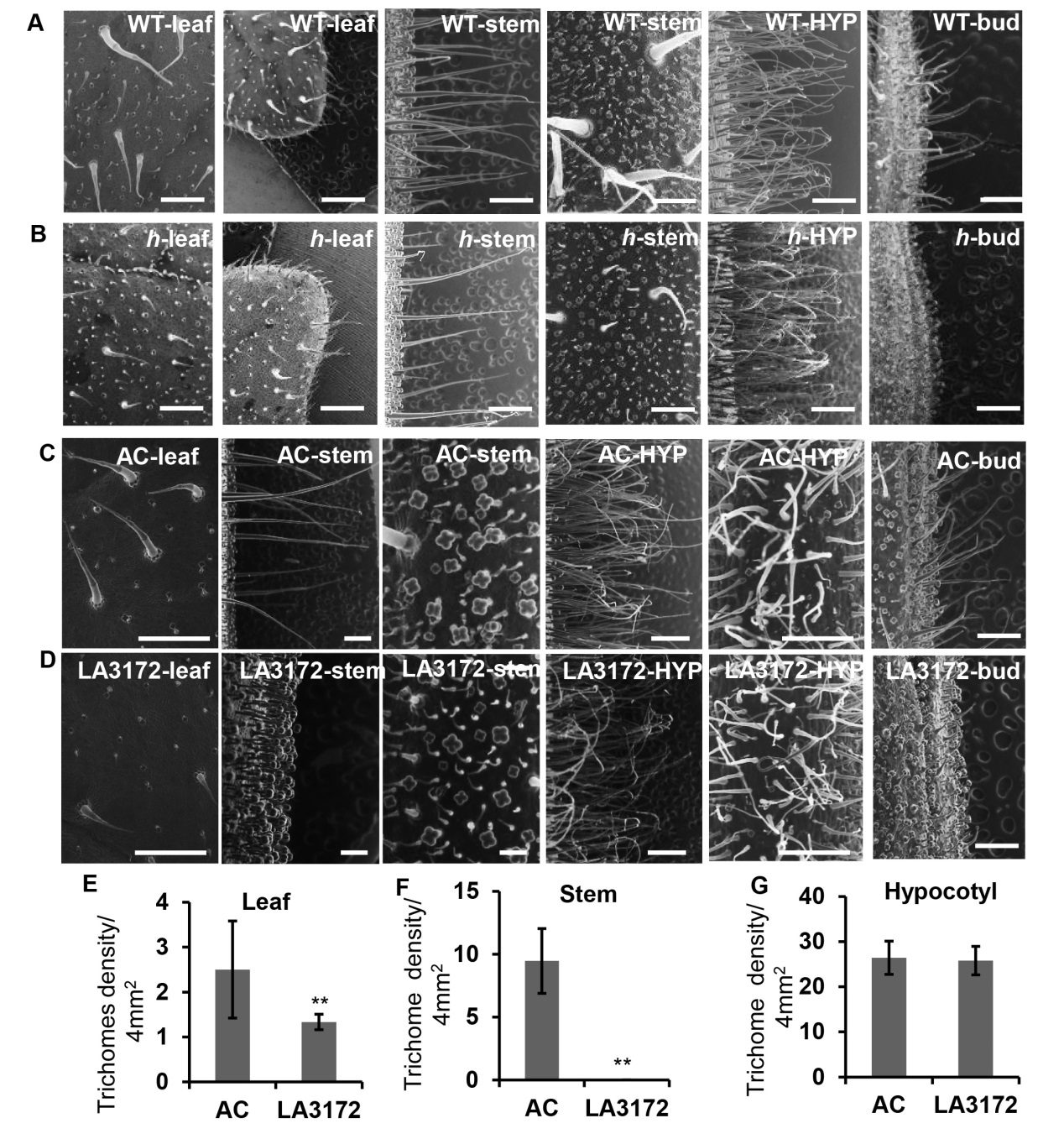
**

**Supplemental Fig. 3 The phenotype of WT, *h* mutant, AC, and LA3172 under SEM.**

(A-D) Trichome phenotype of leaves, stems, hypocotyls (HYP), and buds. (A) WT; (B) *h* mutant; (C) AC; (D) LA3172. Bars (A-D): 1 mm. (E-G) The quantitative analysis of the long trichomes on leaves (E), stems (F), and hypocotyls (G). The error bars represent the standard deviation (SD). Bars annotated with asterisks are significantly different according to Fisher’s LSD test after ANOVA (** P<0.01; * P<0.05).


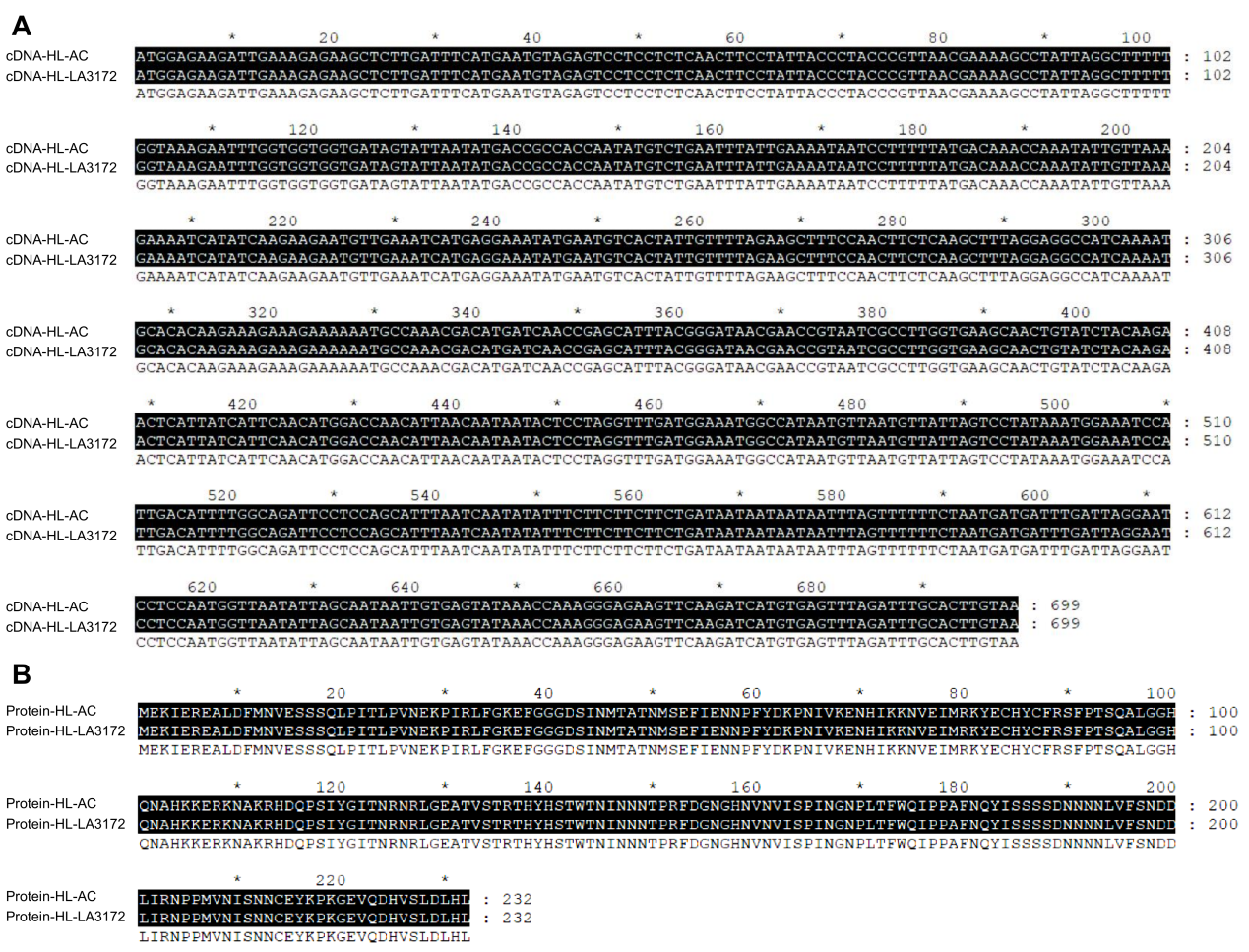


**Supplemental Fig. 4 The alignment of cDNA sequences and protein sequences of HL in AC and LA3172.**

(A) The alignment of HL cDNA sequence in AC and LA3172. (B) The alignment of HL protein sequences in AC and LA3172. The alignments are conducted using Jalview and Genedoc.


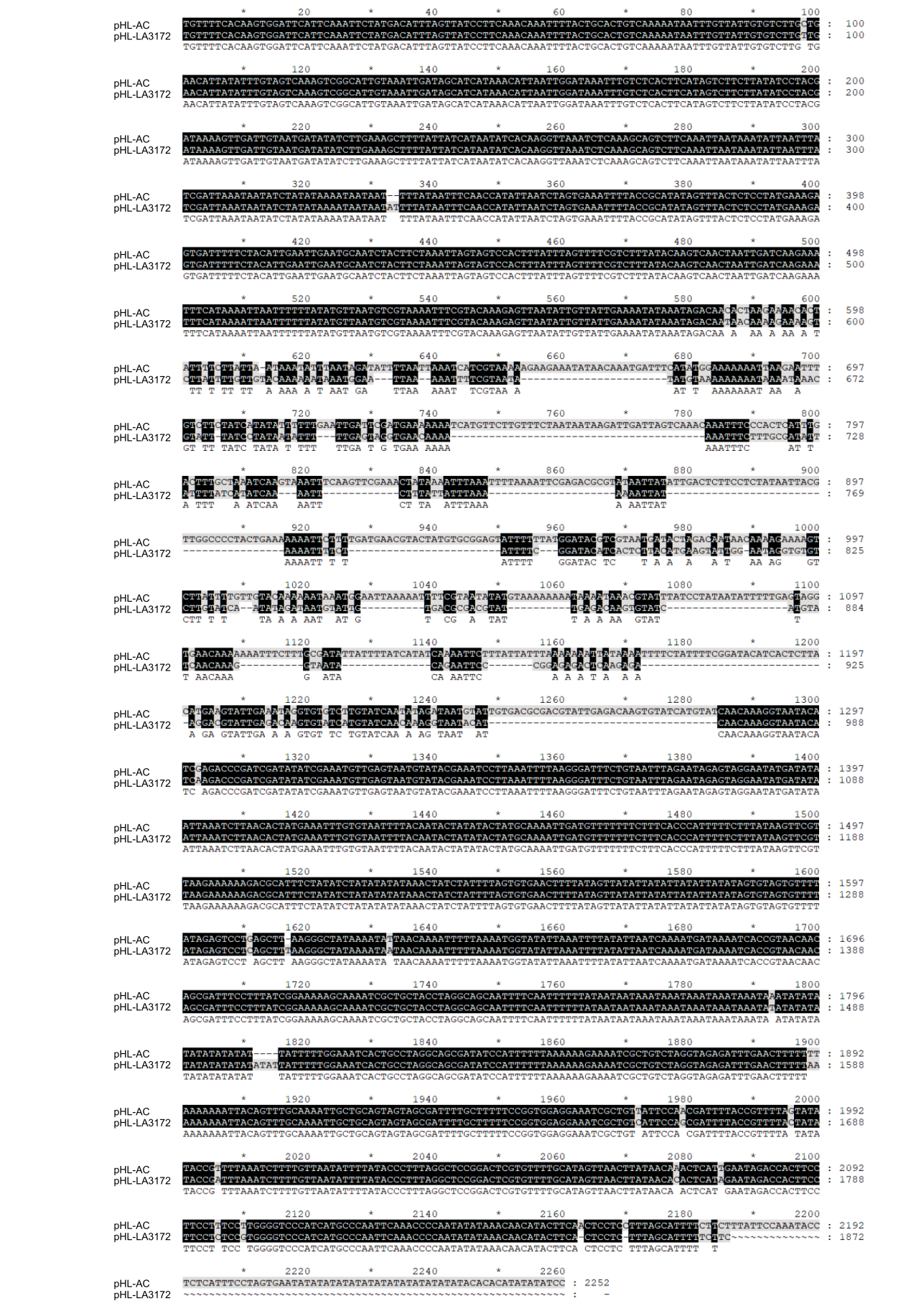


**Supplemental Fig. 5 The alignment of HL promoter in AC and LA3172.**

The alignment is conducted using Jalview and Genedoc.


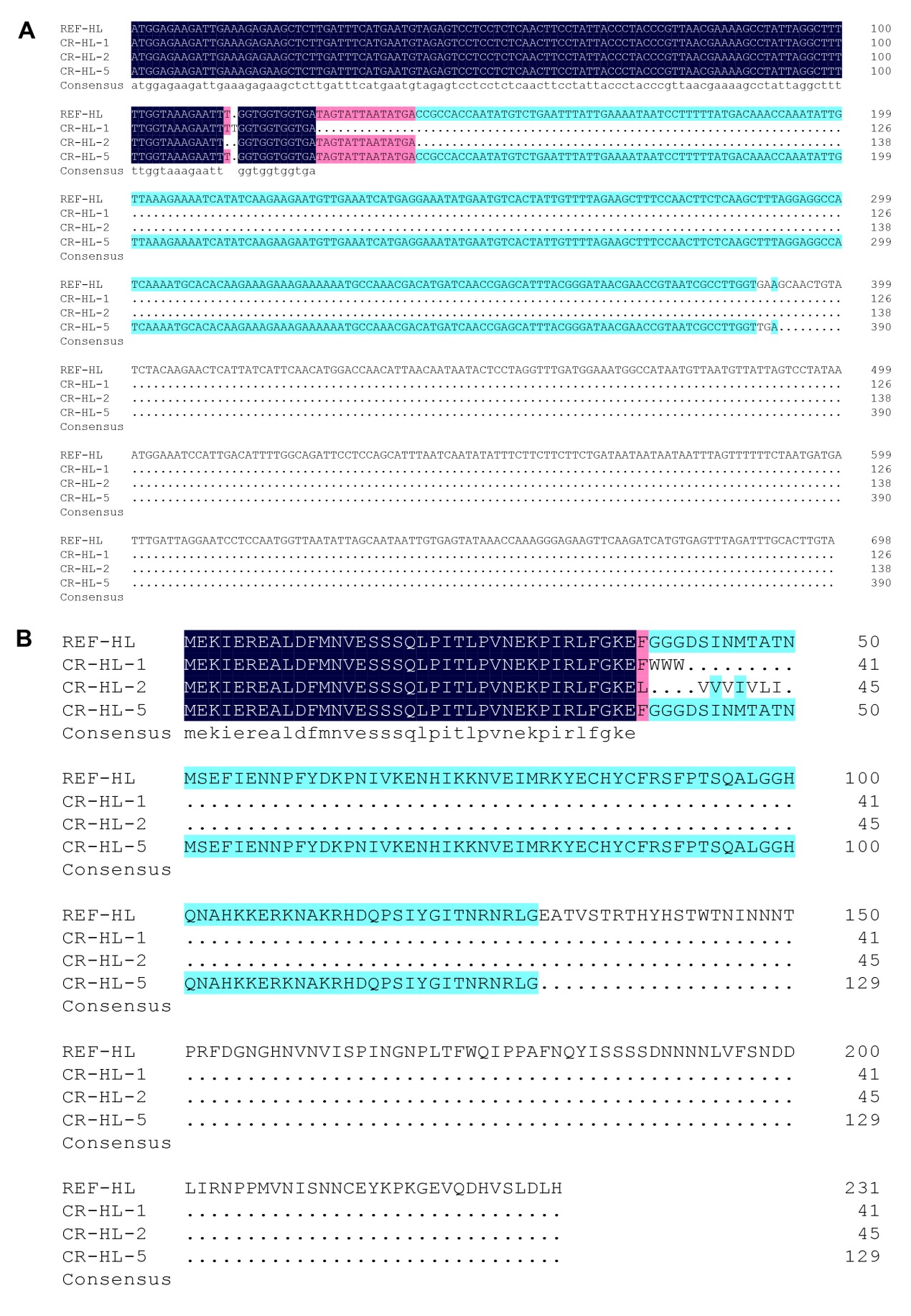


**Supplemental Fig. 6 The sequence analysis of HL-CR lines generated by CRISPR/Cas9.**

(A) CDS alignment of HL in CR-HL plants. (B) CDS alignment of HL in CR-HL plants. The alignment is conducted using DNA MAN.

**
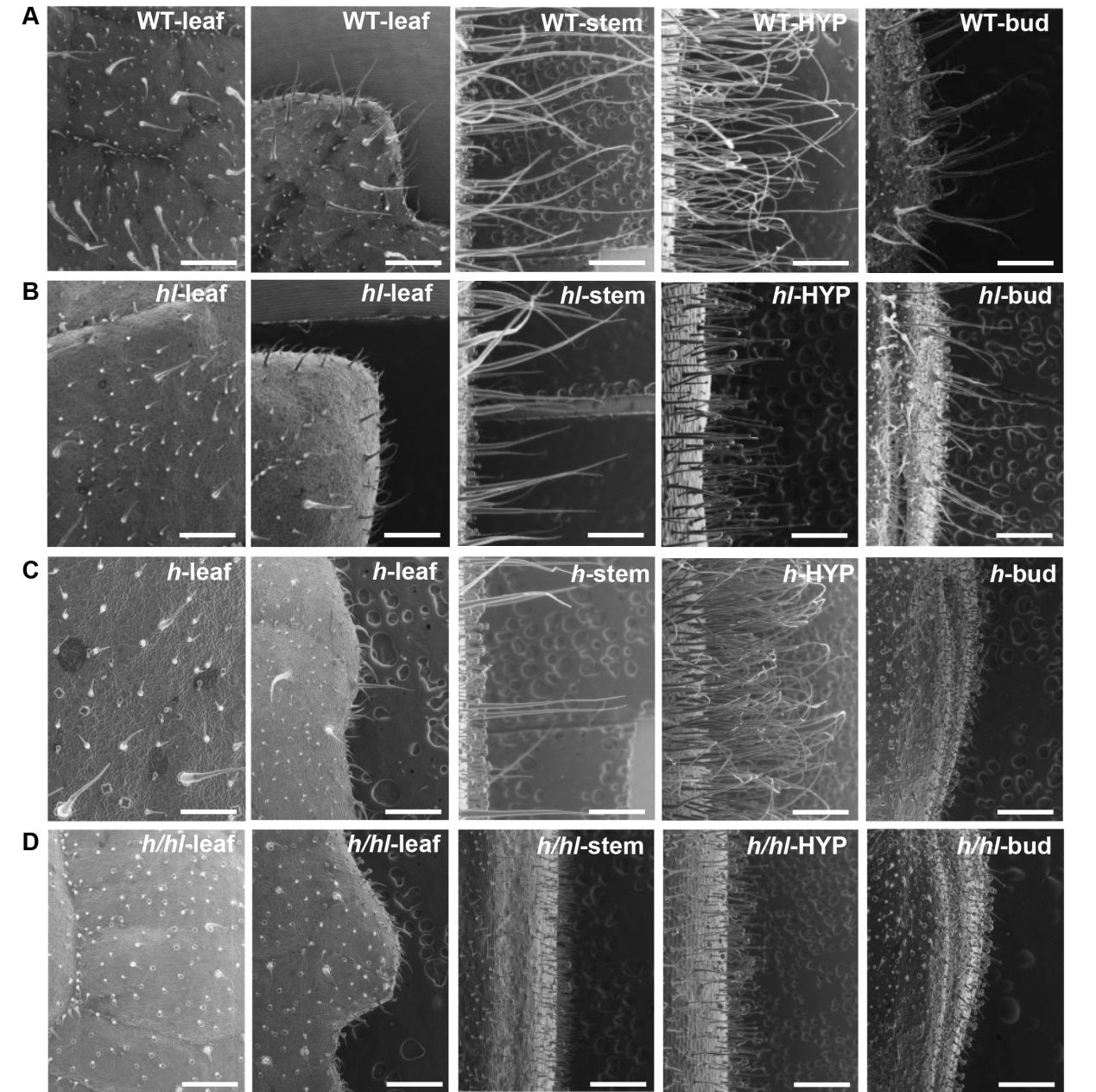
**

**Supplemental Fig. 7 The phenotype of WT, *h* mutant, *hl* mutant, and *h/hl* mutant under SEM.**

(A-D) Trichome phenotype of leaves, stems, hypocotyls (HYP), and sepals. (A) WT; (B) *h* mutant; (C) *hl* mutant; (D) *h*/*hl* mutant. Bars (A-D): 1 mm.


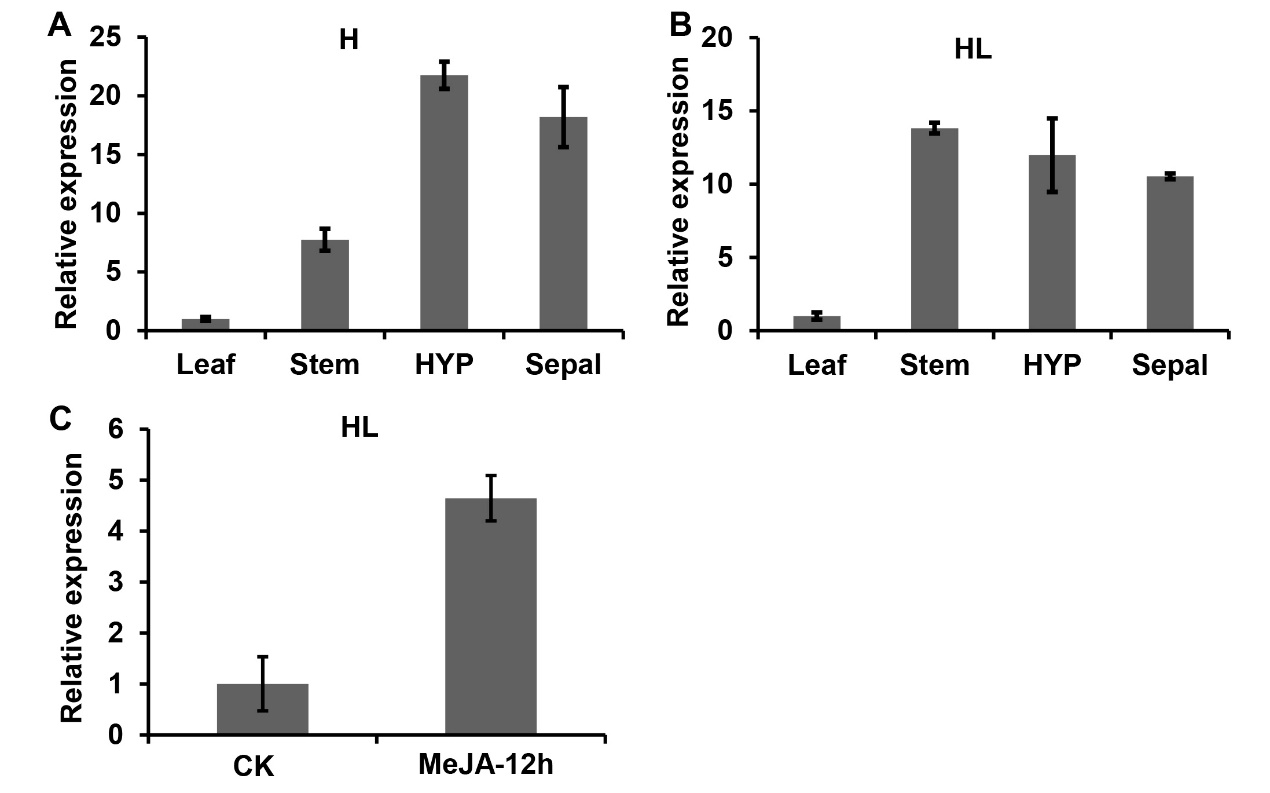


**Supplemental Fig. 8 Relative expression level of *H*/*HL* in different tissues.**

(A-B) The expression of *H* (A) or *HL* (B) shown by qRT-PCR in the leaf, stem, hypocotyl, and sepal. (C) Relative expression level of *HL* is up-regulated after the MeJA treatment for 12h. The error bars represent the standard deviation (SD) of 3 biological replications.


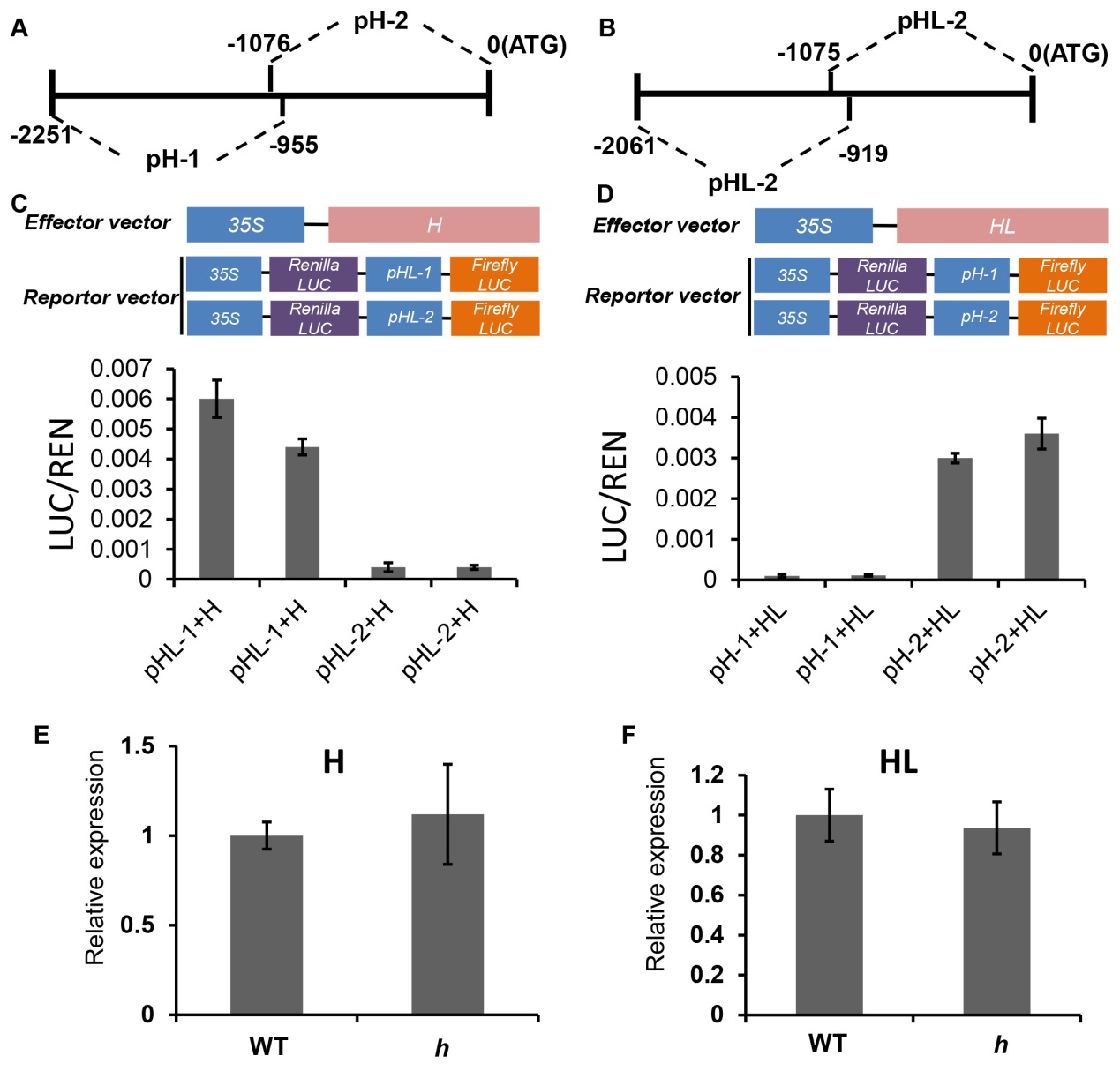


**Supplemental Fig. 9 H and HL have no reciprocal regulation.**

(A) The diagrams of HL promoter used in the dual-LUC assay. The numbers show the location of pHL-1 and pHL-2. (B) The diagrams of H promoter used in the dual-LUC assay. The numbers show the location of pH-1 and pH-2. (C) H does not regulate the expression of *HL* in the dual-LUC assay. The upper graphics show the effector and the reporter vectors. (D) HL does not regulate the expression of *H* in the dual-LUC assay. The upper graphics show the effector and the reporter vectors. (E) qRT-PCR show the expression of *H* has no difference in WT and *hl* mutant. (F) qRT-PCR show the expression of *HL* has no difference in WT and *h* mutant. The error bars represent the standard deviation (SD) of 3 biological replicates.


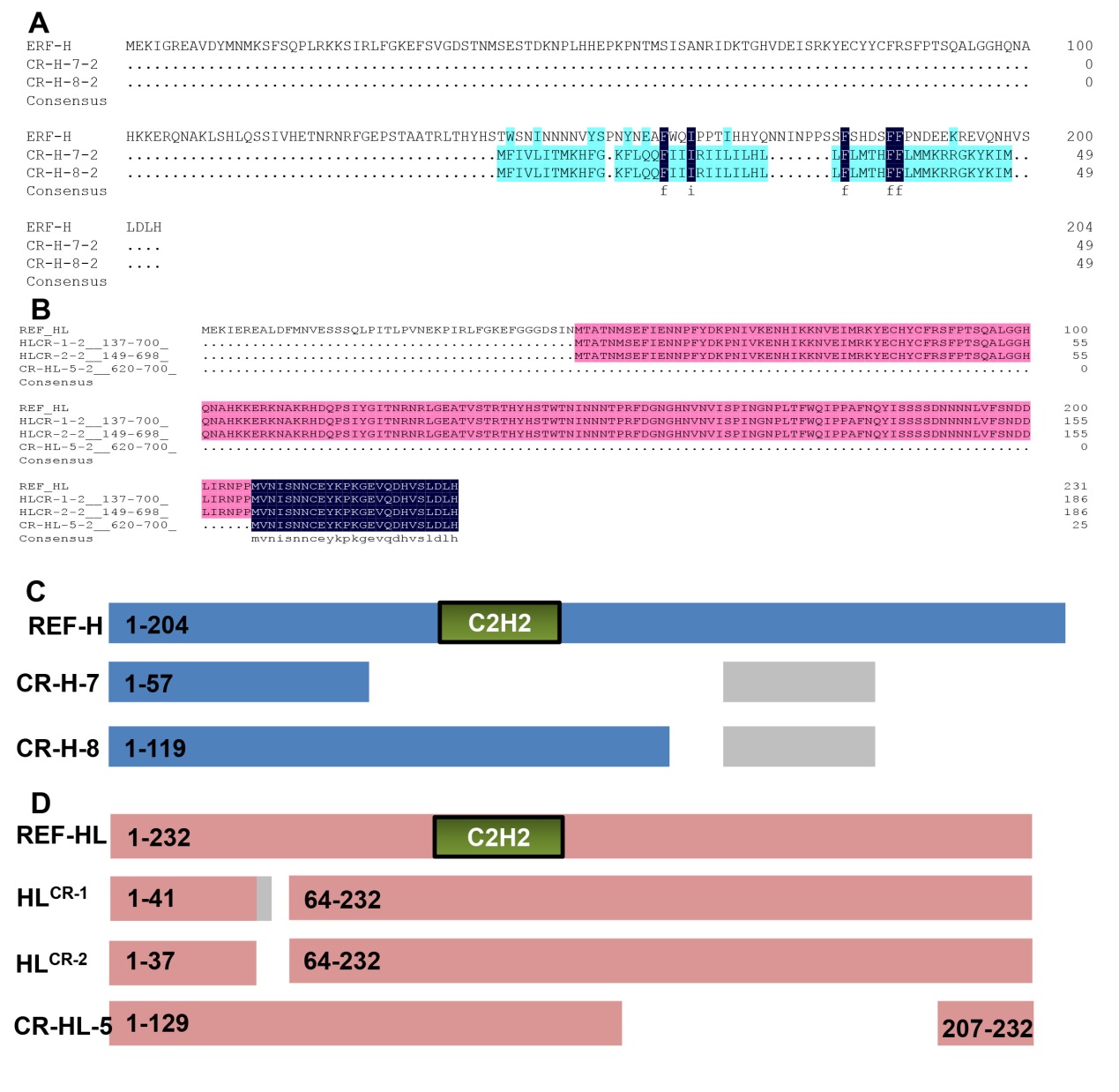


**Supplemental Fig. 10 The alignment of protein sequences.**

(A-B) Protein sequence alignment of C-terminal of H protein in CR-H lines (A) and HL protein in CR-HL lines (B). (C-D) Schematic diagram of protein sequences. The gray boxes show the sequence that cannot be aligned with the reference sequence. The green boxes show C2H2 domain.


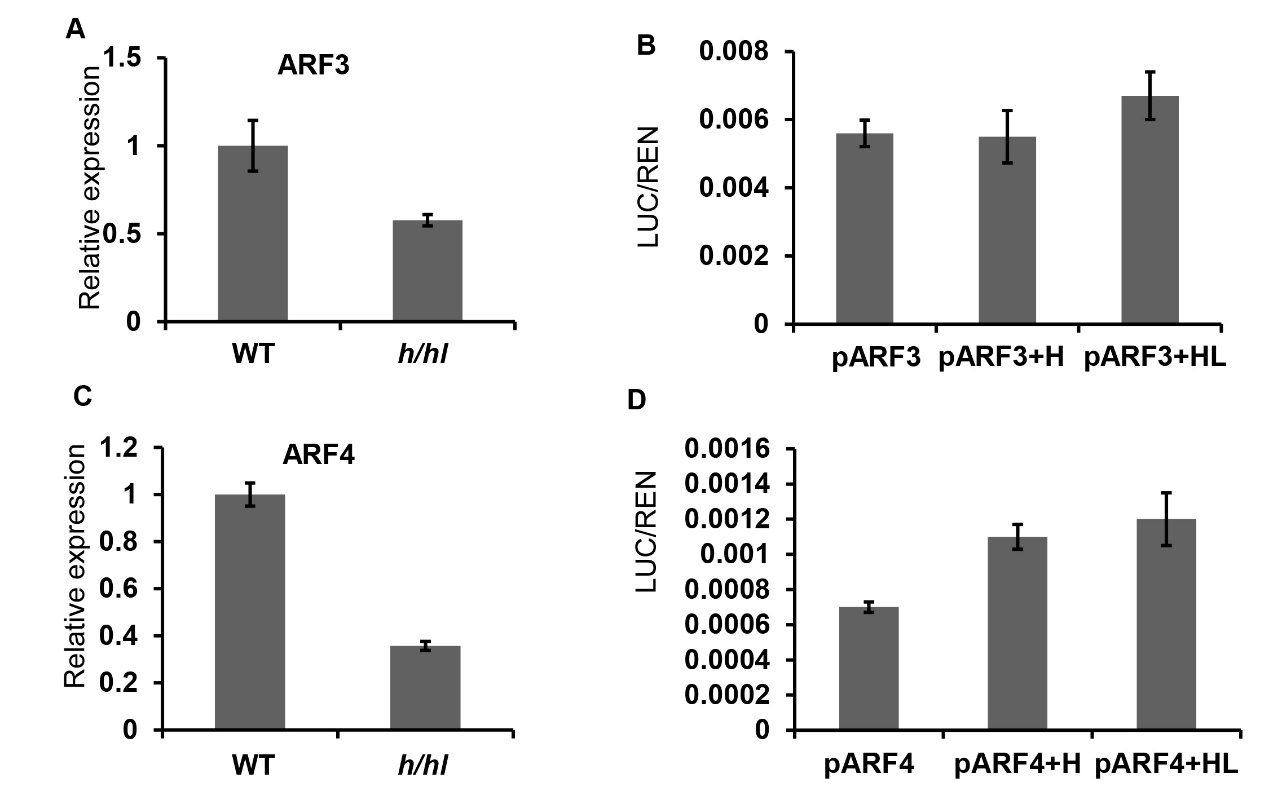


**Supplemental Fig. 11 H and HL regulate the expression of ARF3 and ARF4.**

(A, C) qRT-PCR show the transcription level of ARF3 (A) and ARF4 (C) decreased in *h/hl* mutant than WT. (B, D) Dual-LUC assay show H and HL fail to activate the expression of ARF3 (B) and ARF4 (D). The error bars represent the standard deviation (SD) of 3 biological replicates.
